# The effect of berberine on insulin resistance in women with polycystic ovary syndrome: detailed statistical analysis plan (SAP) for a multicenter randomized controlled trial

**DOI:** 10.1186/s13063-016-1633-5

**Published:** 2016-10-21

**Authors:** Ying Zhang, Jin Sun, Yun-Jiao Zhang, Qian-Yun Chai, Kang Zhang, Hong-Li Ma, Xiao-Ke Wu, Jian-Ping Liu

**Affiliations:** 1 Center for Evidence-based Chinese Medicine, Beijing University of Chinese Medicine, Beijing, 100029 China; 2 Department of Obstetrics and Gynecology, First Affiliated Hospital of Heilongjiang University of Chinese Medicine, Harbin, 150040 China

**Keywords:** Statistical analysis plan, Berberine, Polycystic ovary syndrome, Randomized controlled trial, Traditional Chinese medicine

## Abstract

**Background:**

Although Traditional Chinese Medicine (TCM) has been widely used in clinical settings, a major challenge that remains in TCM is to evaluate its efficacy scientifically. This randomized controlled trial aims to evaluate the efficacy and safety of berberine in the treatment of patients with polycystic ovary syndrome. In order to improve the transparency and research quality of this clinical trial, we prepared this statistical analysis plan (SAP).

**Methods:**

The trial design, primary and secondary outcomes, and safety outcomes were declared to reduce selection biases in data analysis and result reporting. We specified detailed methods for data management and statistical analyses. Statistics in corresponding tables, listings, and graphs were outlined.

**Discussion:**

The SAP provided more detailed information than trial protocol on data management and statistical analysis methods. Any post hoc analyses could be identified via referring to this SAP, and the possible selection bias and performance bias will be reduced in the trial.

**Trial registration:**

This study is registered at ClinicalTrials.gov, NCT01138930, registered on 7 June 2010.

## Update

This paper provides the detailed statistical analysis plan (SAP) for “The effect of berberine on insulin resistance randomized, placebo-controlled trial,” comparing glucose disposal rate (GDR) at week 12. Secondary endpoints and GDR at other time points will provide exploratory results.

## Background

Polycystic ovary syndrome (PCOS) is an ovarian disorder characterized by hyperandrogenism, ovulatory dysfunction, and polycystic ovaries [1]. It is the most common endocrinopathy affecting women of reproductive age, and has attracted a lot of public attention for its relative high prevalence both in communities and clinical settings [2, 3]. Berberine is the major active component of Rhizoma Coptidis, with a broad array of pharmacological effects [4]. Recently, several studies have shown that berberine has positive effects on type 2 diabetes mellitus, insulin resistance, lipid metabolism, nitric oxide production, and the metabolic syndrome [5–8]. This study is a multicenter, double-blind, randomized, placebo-controlled clinical trial. The purpose is to compare the insulin resistance in PCOS patients with and without the use of berberine. This study has been approved by the Ethics Committee of the First Affiliated Hospital Heilongjiang University of Chinese Medicine (2010HZYLL-012) and informed consent was obtained from each patient before any study procedures were performed. Full details of the trial design of this randomized controlled trial (RCT) are given in the study protocol which has been registered and published [9]. Patients will be enrolled at four hospitals in mainland China as stated in the protocol.

Despite Traditional Chinese medicine (TCM) having been increasingly used in clinical settings, a major challenge in TCM is to evaluate its efficacy scientifically [10]. The results of several studies have indicated the low quality of RCTs in TCM [11, 12]. For example, a recent study on bias of risk and outcome-reporting in RCTs revealed inconsistencies between information from trial registration and subsequent publications [13]. The inconsistencies occurred mostly in statistical methods, sample size, primary outcomes, safety reporting, and outcome assessor blinding.

In the study protocol we detailed the overall design and approaches. For a clinical trial, bias may also be introduced in the post hoc selection of data management procedures and the statistical analysis methods. The SAP will provide more transparent information for data analysis methods than the protocol. Besides, the possible dispute and queries can be resolved by the published SAP when publishing trial results. These strategies and practices are important to improve the scientific validity in the clinical research of Chinese Medicine interventions. In order to improve the transparency and research quality of our trial, we prepared this SAP.

### Inclusion criteria

1. Women aged between 18 and 35 years
2. Confirmed diagnosis of PCOS according to the modified Rotterdam criteria, and all patients must be anovulatory plus have either polycystic ovaries and/or hyperandrogenism
3. Body Mass Index equal to or greater than 23 kg/m^2^
4. Women must not be pregnant and not expect to become pregnant within 6 months

### Exclusion criteria

1. Patients who are treated within the past 3 months with other medications known to affect reproductive function or metabolism, including oral contraceptives, gonadotropin- releasing hormone (GnRH) agonists and antagonists, antiandrogens, gonadotropins, antiobesity drugs, Chinese herbal medicines, antidiabetic drugs, such as metformin and thiazolidinediones, somatostatin, diazoxide, angiotensin-converting enzyme (ACE) inhibitors, and calcium channel blockers
2. Patients with other endocrine disorders including 21-hydroxylase deficiency, hyperprolactinemia, uncorrected thyroid disease, or suspected Cushing’s syndrome
3. Patients with known severe organ dysfunction or any mental illness

### Sample size

It is assumed that women with PCOS would have an insulin resistance comparable to women with type 2 diabetes. Based on a previous study [4], GDR was 7.42 ± 2.37 mg/(kg × min) in the berberine group and 6.06 ± 2.21 mg/(kg × min) in the control group. To detect this difference between the two arms, the sample size required was 52 per group, given a two-sided *α* = 0.05 (type I error) and *β* = 0.1 (type II error).

### Randomization and allocation concealment

Eligible participants had been randomized into each of the two arms: berberine (0.5 g, three times per day) or placebo. Berberine or placebo medications were administrated orally for 12 weeks. Patients, investigators, and physicians were blind to the assignment.

### Data management

From the beginning of this study, a Data Management Group has been formed (J Sun, YJ Zhang, K Zhang). They were involved in setting up studies, collecting or entering data, cleaning data, and managing accrual data until the study could be considered ready for analysis. The data in Case Report Form (CRF) all came from the original files and the consistencies between the two were monitored periodically. Data should be timely, accurately, and integrally and veritably filled in the CRF. The web-based data management system was also available at: http://www.medresman.org/login.aspx.

### Guidelines for analysis

This SAP aims to detail the presentation and analysis methods for the main paper(s) reporting results of this study. Therefore, biases related to selective reporting from our study could be reduced. International Council on Harmonization (ICH) guidance on statistical principles for clinical trials (E9) [14] and the Consolidated Standards of Reporting Trials (CONSORT) Statement [15] for the reporting results were followed.

### Statistical analysis set

#### Intention-to-treat set

The intention-to-treat (ITT) set is defined as all patients in the intervention (berberine or placebo) arms to which they were randomized, regardless of the availability of data at follow-up, and the real intervention that they received during the period of the trial. Patients will be included in the group to which they were randomized.

The ITT analysis will be reported mainly for efficacy evaluation of primary and secondary outcomes. In addition, ITT will be used in the balance testing of demographic data, disease history, and laboratory examinations at baseline.

#### Per-protocol set

The per-protocol set (PPS) includes patients who have completed prescribed treatments in the whole trial period without severe protocol violation.

The severe protocol violations will include (but not be limited to) the following conditions:

1. Not meeting the inclusion criteria
2. The use of concomitant medication which may add confounding to the estimation of efficacy and safety
3. Being beyond the time window, loss to follow-up, or dropout

Any patients with varying degrees of protocol violation will be confirmed in the blinded data review meeting.

Efficacy analyses will be carried out for both ITT and PPS. If the results of the ITT and PPS are inconsistent, sensitivity analyses will be conducted, and possible causes will be investigated. Subgroup analyses by patients’ characteristics, such as age, weight, Body Mass index, waist/hip circumference ratio, etc. will be carried out.

#### Safety set

The safety set (SS) includes the patients who received at least one-time medication treatment. The SS is the main set of safety assessment in this trial.

### Baseline demographic characteristics

Discrete variables will be summarized by frequencies and percentages. Continuously distributed variables will be summarized using either mean ± standard deviation (SD) for data with normal distribution, or median and interquartile range for nonnormally distributed data.

### Primary outcome and hypothesis

The primary outcome is the change of GDR from baseline to week 12. The GDR is defined as the amount of glucose required to maintain stable blood glucose concentrations during the last 30 min of the hyperinsulinemic-euglycemic clamping. The mean value of change (*μ*), will be computed. The relative efficacy of berberine on increasing GDR compared to placebo will be tested at the one-sided significance level of 0.025 under the null hypothesis and alternative hypothesis as below:

- H_0_: *μ*_*Berberin*e_ = *μ*_*Placebo*,_
- H_1_: *μ*_*Berberine*_ > *μ*_*Placebo*._

Statistics relevant to the primary outcome, including eligible number, mean, SD, median, interquartile range, and minimum and maximum, will be calculated. A paired Student’s *t* test/signed rank test will be used to compare the difference between baseline and post treatment within each group. The Analysis of Covariance (ANCOVA) model will be constructed in estimating the difference of GDR between baseline and post treatment. Covariates will be the baseline value of GDR considering the effects of different sites and groups. Based on this model, we will calculate least squares means (LSMEANS) of the difference accompanying 95 % confidence interval between the two groups. To explore the consistency of results across centers, the interaction between treatment effect and centers or groups will be added into the model. The interaction effect will be considered statistically significant when the *P* value for the interaction test is equal to or less than 0.10. If the interaction is statistically significant, further descriptive investigation will be conducted in subgroups by sites.

### Secondary outcomes and statistical methods

The secondary outcomes will be analyzed based on both ITT and PPS:

1. Oral glucose tolerance test (OGTT): serum for glucose, insulin, and c-peptide levels will be determined.
  The outcomes of the OGTT will be analyzed by the *t* test/Wilcoxon rank sum test. The trapezoidal method will be applied to calculate the area under the curve (AUC) of OGTT values at different time points [16] using the following formula:

$$ \left({X}_{0 \min }+{X}_{180 \min}\right)/2 + {X}_{30 \min }+{X}_{60 \min }+{X}_{120 \min }. $$

2. Ovarian androgen biosynthesis as measured by human chorionic gonadotropin (hCG), stimulated production of 17-hydroxyprogesterone (17-OHP), androstedione (A2), and testosterone (T) levels
3. Hormonal profile including: testosterone, sex hormone-binding globulin (SHBG), follicle-stimulating hormone (FSH), luteinizing hormone (LH), and dehydroepiandrosterone sulfate (DHEAS) levels
4. Fasting lipid metabolic profile: cholesterol, triglycerides (TG), high-density lipoprotein cholesterol (HDL-C), and low-density lipoprotein cholesterol (LDL-C) levels
5. Weight, waist/hip circumference ratio, blood pressure, Ferriman-Gallwey Score, and severity of acne before and after treatment

Student’s *t* test or the Wilcoxon rank sum test will be used to test the difference of the above outcomes between groups according to the distribution of variables.

### Safety outcomes and statistical methods

Safety outcomes, including vital signs, adverse events, and renal and liver function (including three categories: normal, abnormal without clinical significance, and abnormal with clinical significance), will be analyzed based on the SS.

Shift tables will be constructed to map the changes of abnormal renal and liver function test results in each visit. The vital signs comparison between groups at baseline and post treatment and the changes relative to baseline will be described with mean, SD or median and interquartile range and Student’s *t* test or the Wilcoxon rank sum test will be used accordingly. Adverse events will be listed separately by type (nonserious adverse events, serious adverse events), by visit, and by real intervention.

### Handling of missing data

The imputation will be carried out for primary and secondary outcomes only. When analyzing the efficacy in ITT, the Last Observation Carried Forward (LOCF) imputation method will be used.

### Interim analysis

No interim analysis was planned. All results and conclusions will be derived from the final analysis of this trial.

### CONSORT flow diagram

The profile of patients will be summarized in a CONSORT flow diagram.

## Abbreviations

17-OHP: 17-hydroxyprogesterone
A2: Androstedione
ACE: Angiotensin-converting enzyme
ANCOVA: Analysis of Covariance
AUC: Area under the curve
CONSORT: Consolidated Standards of Reporting Trials
CRF: Case Report Form
DHEAS: Dehydroepiandrosterone sulfate
FSH: Follicle-stimulating hormone
GDR: Glucose disposal rate
GnRH: Gonadotropin-releasing hormone
hCG: Human chorionic gonadotropin
HDL-C: High-density lipoprotein cholesterol
ICH: The International Council for Harmonization
ITT: Intention-to-treat
LDL-C: Low-density lipoprotein cholesterol
LH: Luteinizing hormone
LOCF: Last Observation Carried Forward
LSMEANS: Least mean squares
OGTT: Oral glucose tolerance test
PCOS: Polycystic ovary syndrome
PPS: Per-protocol set
RCT: Randomized controlled trial
SAP: Statistical analysis plan
SD: Standard deviation
SHBG: Sex hormone-binding globulin
SS: Safety set
T: Testosterone
TCM: Traditional Chinese Medicine
TG: Triglycerides
